# Quadricuspid Aortic Valvulopathy and Acute Type A Aortic Dissection

**DOI:** 10.1055/s-0039-1692457

**Published:** 2019-10-16

**Authors:** Sheila L. Klassen, Stuart J. Hutchison

**Affiliations:** 1 Department of Cardiac Sciences, Libin Cardiovascular Institute of Alberta, University of Calgary, Alberta, Canada

**Keywords:** quadricuspid aortic valve, aortic dissection, aortopathy, ascending aorta

## Abstract

This case report describes a 55-year-old male who presented with acute Type A aortic dissection. He underwent emergent surgical repair, and his intraoperative transesophageal echocardiography revealed a quadricuspid aortic valve. His aortic root measured 45 mm. Quadricuspid aortic valves have previously been associated with aortic root dilation. This case illustrates the possible association of quadricuspid aortic valves with aortic dissection, similar to what is described with bicuspid valves.

## Introduction

While congenital bicuspid aortic valves are well known to be associated with aortopathy and its complications including dissection of the ascending aorta, congenital quadricuspid aortic valves are much less common and less well understood. We present a case which illustrates the possible association of quadricuspid aortic valves with aortic dissection, similar to what is described with bicuspid valves.

## Case Presentation

A 55-year-old male presented to the emergency department with abrupt onset of severe chest pain, radiating through to the back, which had started 12 hours earlier and remained severe. There was no associated dyspnea, presyncope, syncope, or neurologic deficit.

He had been diagnosed with mild hypertension 1 month ago but not yet started on pharmacotherapy. He was a 15 pack-year smoker. He had no known cardiac disease, no history of chest pain, no Marfanoid features, no family history of aortic disease, no prior traumatic injury, and no systemic complaints.

On examination, he was distressed and diaphoretic with ongoing pain. His blood pressure was 175/90 mm Hg, and the heart rate was 90 bpm. There were no pulse deficits. There was a 2/6 systolic ejection murmur at the base of the heart. No pericardial friction rub was present. Jugular venous pressure was normal.

The 12-lead electrocardiogram showed normal sinus rhythm. The anteroposterior chest radiograph revealed a normal-sized heart but an abnormal aortic contour in the ascending and arch locations. There was no left pleural effusion and no intimal displacement sign at the distal aortic arch.

**Fig. 1:**
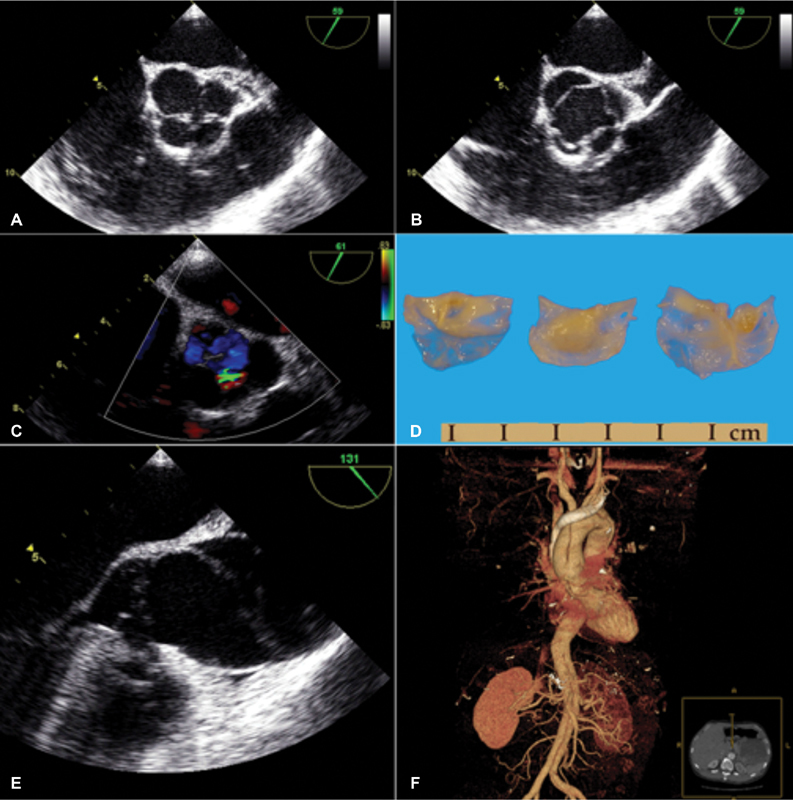
( **A, B** ) transesophageal echocardiography (TEE) cross-sectional view of the aortic valve revealing four cusps, with slight inequality of size: diastole—left, systole—right. ( **C** ) TEE image with color Doppler flow mapping revealing an aortic insufficiency jet arising from the smaller cusps. ( **D** ) pathology image displaying the larger cusps, and the smaller cusps as one piece of tissue on the right. One of the smaller cusps is prominently thickened and fenestrated, as is the larger cusp in the middle. ( **E** ) TEE view of the dilated ascending aorta and intimal flap extending to 1 cm above the posterior sinotubular junction. ( **F** ) three-dimensional computed tomography volume reconstruction of the aorta depicting the intimal flap starting in the ascending aorta and extending down into the left iliac artery.

A contrast-enhanced computed tomography scan was obtained to assess the possibility of acute aortic dissection. An intimal flap was imaged beginning in the ascending aorta, which was 45 mm in diameter, and continuing around the arch, down the descending aorta and extending into the left iliac artery ( Fig. 1 ). Transthoracic echocardiography revealed moderate aortic valve insufficiency.

The patient underwent surgical repair with graft replacement of the ascending aorta and mechanical aortic valve replacement. Intraoperative transesophageal echocardiography revealed that the aortic valve was quadricuspid, which was confirmed at surgical inspection. The intimal tear was located above the noncoronary cusp. The pattern of the quadricuspid valve was of two larger and two smaller cusps ( Fig. 1 ). Pathological examination revealed that the smaller cusps were thickened, and there were fenestrations. The patient was discharged on the 7th postoperative day, on warfarin and metoprolol, and remains well in follow-up.

## Discussion

Congenital quadricuspid aortic valvulopathy occurs with an incidence of 0.01%. 1 It is slightly more common in males (male:female ratio 1.6:1), and the etiology is theorized to be abnormal septation of the truncus arteriosus or abnormal endocardial cushion formation. 2 The Hurwitz & Roberts classification system divides quadricuspid aortic valves into seven types classified from A to G. Type A (four equal-sized cusps), Type B (three equal-sized cusps and one smaller cusp), and Type C (two equal-larger cusps and two equal smaller cusps) are most often described. 3 Dilation of the ascending aorta has been described 2 4 with a frequency of 29% in a case series by Tsang et al. 5 In this case series of 50 patients without associated history of hypertension, dilation involved the aortic root and ascending aorta with equal incidence and the majority of those with a dilated aorta also had moderate or worse aortic insufficiency. Their case series did not report any aortic dissection over a mean 5-year follow-up period. Aortic dilation has been previously described when morphology of the aortic cusps is found to be Type B. 2 Quadricuspid aortic valves are associated with aortic insufficiency due to malcoaptation of the leaflets 2 in up to 90% of cases 5 and abnormal shear stresses on the leaflets. 3 In one small case series, five of nine patients with quadricuspid aortic valve and aortic insufficiency requiring valve replacement had a fenestrated cusp. 4 The most frequently associated disorder is coronary artery and coronary ostium abnormalities which may be present in up to 10% of cases. 6 Echocardiography is the most common mode of detection of quadricuspid aortic valves, though cardiac computed tomography carries the additional benefit of defining anatomy of the coronary ostia. 7

To the best of our knowledge, there has not yet been a report of quadricuspid aortic valves associated with acute aortic dissection. In our case, the modest degree of hypertension was recorded when the patient was in severe pain from aortic dissection. Additionally, the ready control of blood pressure with pain control and the lack of a substantial history of hypertension argues against hypertension as a cause of aortic dissection in this case and suggests underlying aortic aortopathy associated with the quadricuspid aortic valve.
